# Toll-like receptor 4 deficiency in mice impairs venous thrombus resolution

**DOI:** 10.3389/fmolb.2023.1165589

**Published:** 2023-05-12

**Authors:** Haixin Yuan, Xiaoxi Huang, Jie Ding

**Affiliations:** Medical Research Center, Beijing Chao-Yang Hospital, Capital Medical University, Beijing, China

**Keywords:** TLR4, venous thrombus resolution, mice, macrophages, NF-κB

## Abstract

**Objective:** Toll-like receptor 4 (TLR4) is crucial to the development of sterile inflammatory responses. The deep venous thrombosis resolution (DVT) is similar to sterile inflammation, so we hypothesize that TLR4 is involved.

**Methods and Results:** We evaluated the effects of TLR4 deficiency on thrombus lysis *in vivo*, and explored the mechanisms *in vitro*. DVT mouse model was established by inferior vena cava (IVC) ligation. After the IVC ligation (1, 3, and 7 d), the mice were euthanized to collect the venous thrombus. The Tlr4−/− mice had significantly elevated weight/length ratios of thrombi at 3 and 7 d and increased collagen content at 3 d after IVC ligation, in addition to significantly lesser intrathrombus infiltration of neutrophils and macrophages, lower monocyte chemoattractant protein-1 (MCP-1) and matrix metalloproteinase-9 (MMP-9) expression in thrombus tissue sections and homogenates, and lower pro-MMP-9 activity at 3 d after IVC ligation than wild-type mice. After 7 days of IVC ligation, VEGF, IFNβ, and MCP-5 protein expression were decreased in venous thrombus from Tlr4−/− mice. 2 ml of 3% thioglycolate was injected intraperitoneally and peritoneal exudate was collected 3 days later from Tlr4−/− and wild type mice respectively. The intraperitoneal macrophages were isolated from adherent culture after centrifugation. Lipopolysaccharide (LPS) can activate TLR4/NF-κB signalling pathway in a concentration-dependent manner, initiated p65 nuclear translocation, IκBα phosphorylation and degradation, MMP-9 and MCP-1 transcription in WT intraperitoneal macrophages but not in Tlr4−/− intraperitoneal macrophages.

**Conclusion:** TLR4 is involved in venous thrombosis resolution through NF-κB pathway. Loss of TLR4 in mice impairs the process.

## Introduction

Deep venous thrombosis (DVT) and pulmonary thromboembolism (PTE) represent the same dynamic disease known as venous thromboembolism (VTE) but occur at different locations. DVT resolution is an inflammation process (Colling et al., 2021). Natural thrombus resolution is characterized by tissue organization that resembles the processes of wound healing. Leukocytes tend to invade the thrombus in a specific sequence, suggesting that they play important roles in thrombus resolution. Neutrophils have been detected in the thrombus at early stages of DVT resolution and macrophages are detected at later stages. Neutropenia impairs DVT resolution in a rat stasis DVT model, most likely by altering normal fibrinolysis and collagenolysis (Varma et al., 2003). Monocyte influx into the thrombus occurs following thrombogenesis, which is accompanied by elevated MCP-1 concentrations. In a stasis DVT rat model, both peritoneal macrophage administration and MCP-1 injection enhanced thrombus resolution (Ali et al., 2006).

TLR4 participates in innate immunity by recognizing pathogen-associated molecular patterns to initiate pro-inflammatory signaling pathways (Akira and Takeda, 2004; Anthoney et al., 2018). TLR4 is activated by either bacterial endotoxin or endogenous ligands that trigger the formation of the activated homodimers (E-MD-2-TLR4) 2. During the innate immune response, Myeloid differentiation factor 88 (MyD88) and the Toll/IL-1 receptor (TIR) of TLR4 interact to activate the nuclear factor-kappa B (NF-κB) pathway, resulting in the production of cytokines, chemokines, and other effector molecules.

DVT resolution is considered to be a sterile inflammatory process (Wakefield et al., 2008). We therefore hypothesised that this process induces localised production of endogenous ligands, which promotes the TLR4 signalling pathway to participate in DVT resolution. To assess the pathological role of TLR4 in DVT resolution *in vivo*, we examined the length and weight of venous thrombus in wild-type and TLR4-deficient mice, and measured expression of cytokines and chemokines in thrombus homogenates. To study the involvement of the TLR4/NF-κB signalling in DVT resolution *in vitro*, we detected IκBα phosphorylation, p65 nuclear translocation and transcription of MCP-1, MMP-9 and urokinase plasminogen activator (uPA) in peritoneal macrophages from TLR4-intact and TLR4-deficient mice.

## Materials and methods

### Animals

The C57BL/10ScNJ strain as TLR4-deficient mice are homozygous deletion in all three exons of the TLR4 locus (Poltorak et al., 1998). C57B/10J strain were used as control mice with intact TLR4. The Center for Model Animal Research at Nanjing University provided the mice (Nanjing, China). All mice were kept in clean animal house of the specific pathogen-free grade at Experimental Animal Department, Capital Medical University. Male mice weighing 25–30 g were used for all studies. The Animal Ethics Committee gave its approval to each animal experiment related to the topic.

### DVT model

As previously described in the literature, the mice were first anesthetized with isoflurane, followed by a 2 cm incision along the abdominal midline. The IVC were separated from artery with a glass needle, and ligated with 5–0 silk threads to prepare thrombus model of venous stasis (Poltorak et al., 1998). Finally, after the IVC ligation (1, 3, and 7 days), the mice were euthanized to collect the venous thrombus when intraperitoneally injected with pentobarbital (50 mg/kg body mass). After separating the thrombus from the venous wall, the length and weight of the thrombus samples were measured. 10 to 15 mice were analyzed in each group. All the animal experiments were repeated three times. The representative results were presented.

### Collagen assay

Thrombotic collagen content was detected using a commercially kits (Bicolor; Carrickfergus, United Kingdom). The collagen amount of each sample was corrected by the thrombus weight (μg·ml^−1^·mg thrombus^−1^).

### Histological analysis

Following IVC ligation, IVC with thrombus was collected on days 1, 3, and 7 and then preserved with 4% paraformaldehyde. Sections with a 4 micron thickness were created from IVC tissues that had been fixed in paraffin. Hematoxylin and eosin staining of tissue slices was carried out according to protocol.

### Immune-histochemical evaluation

Consecutive deparaffenizations of paraffin slices were performed. To minimize non-specific staining, the slices were successively processed with 30% H_2_O_2_ to impede peroxidase activity and 1% bovine serum albumin to obstruct the non-specific reaction. Next, tissue sections were separately covered with the following different antibodies at the most appropriate concentration: anti-MPO (1:200, Abcam, Cambridge, UK), anti-F4/80 (1:100, Abcam, Cambridge, UK), anti-MCP1 (1:200, Abcam, Cambridge, UK), anti-MMP9 (1:100, R&D Systems; Minneapolis, United States), and anti-VEGF (1:200, Abcam, Cambridge, UK). Finally, color development was carried out using a DAB kit after the slice had been co-incubated with the polink-2 plus Rpolymer HRP detection system (Zsbio; Beijing, China). Five high power areas (×400) inside the thrombus were recorded blindly, and the total number of positively stained cells was summed.

### Chemokine/cytokine/VEGF enzyme-linked immunosorbent assay (ELISA)

Thrombus samples were harvested at corresponding times. Proteinase inhibitors (Roche Diagnostics) were added to the phosphate buffer (pH 7.2) to prevent protein degradation. The thrombus was homogenized with 0.3 mL of the buffer. The homogenate was spun at 12,000 g for 15 min at 4°C before the supernatant was removed. A standard BCA kit was applied to determine the amount of protein concentration in the supernatant. Concentrations were normalized to the total protein level. The concentrations of interferon (IFN)β, MCP-5, MMP-9, VEGF (R&D Systems), and uPA (Cell Science) were measured separately using corresponding ELISA kits.

### Gelatin substrate zymoscopy analysis

According to the user’s instructions, a Gelatin-Zymography kit (Applygen; Beijing, China) was utilized to identify the MMP-2 and MMP-9 activity in the thrombus. Using the GEL-Pro Analyzer software version 4.0, band concentrations were calculated (Media Contronetics; Rockville, MD, United States). The MMP activity was indicated by the intensity of the MMP bands in equal amounts of total protein (μg).

### Intraperitoneal macrophages culture

To induce the exudation of intraperitoneal macrophages, 2 mL of 3% thioglycolate (Sigma-Aldrich) was intraperitoneally administered to WT (intact) and TLR4-deficient mice, respectively. After 3 days, peritoneal exudate from mice was collected and then centrifuged for exudate cells. Cells were resuspended in antibiotic-free RPMI 1640 media with 2% FBS after the supernatant had been removed. At 37°C for 2 hours, the resuspended cells were seeded on 6-well cell culture dishes. The culture medium was then used to extract the non-adherent cells. The novel medium was used to cultivate adherent cells, which were identified as macrophages. Cells were extracted after being stimulated with lipopolysaccharide (LPS) at various doses (0, 1, and 10 M) for 2 h in order to conduct a western blot or a quantitative real-time PCR assay.

### Western blotting

Nuclear and cytoplasmic fractions were extracted from macrophages according to the standard protocol (Thermo Fisher; Waltham, MA, United States) for detecting p65 translocation and IκBα phosphorylation. Equal quantities of the protein were combined with loading buffer after the protein concentration was determined using the BCA method, and then the mixture was electrophoresed on an 8% sodium dodecyl sulfate-polyacrylamide gel. The proteins were then transported to a nylon membrane following the completion of the electrophoresis. Bands were detected using an ECL instrument (Amersham Biosciences) after incubation with the optimally diluted primary and secondary antibodies. Bands intensity representing protein content were detected using image analysis software (Image Lab^TM^ Software; Hercules, CA, United States). And the intensities were calculated relative to the lamin A and tubulin reference standards.

### mRNA analysis

Applying RNA extraction kits (Biotech; Beijing, China), total RNA was obtained from primary peritoneal macrophages or venous thrombosis. Reverse the mRNA to cDNA with the Super Mol III M-MLV reverse transcriptase (Biotechnology; Beijing, China). The concentration of the cDNA was determined. With these templates, quantitative real-time PCR was used to quantify the relative expression of the target gene. The internal control was the mouse β-actin gene. Corresponding to each cDNA, a negative control for amplification was included. Using the 2^−(△Ct)^ technique, the relative expression of the target genes was determined.

### Analytical statistics

The mean and standard deviation (SD) are used to express experimental data. Utilizing the Student’s t test, statistical analysis was conducted (SPSS 17.2; Chicago, IL, United States). Using the GraphPad Prism 5 program, graphs of the results were produced. When a difference reached *p* < 0.05, it was deemed statistically significant.

## Results

### Thrombus resolution was compromised in mice lacking TLR4

**FIGURE 1:**
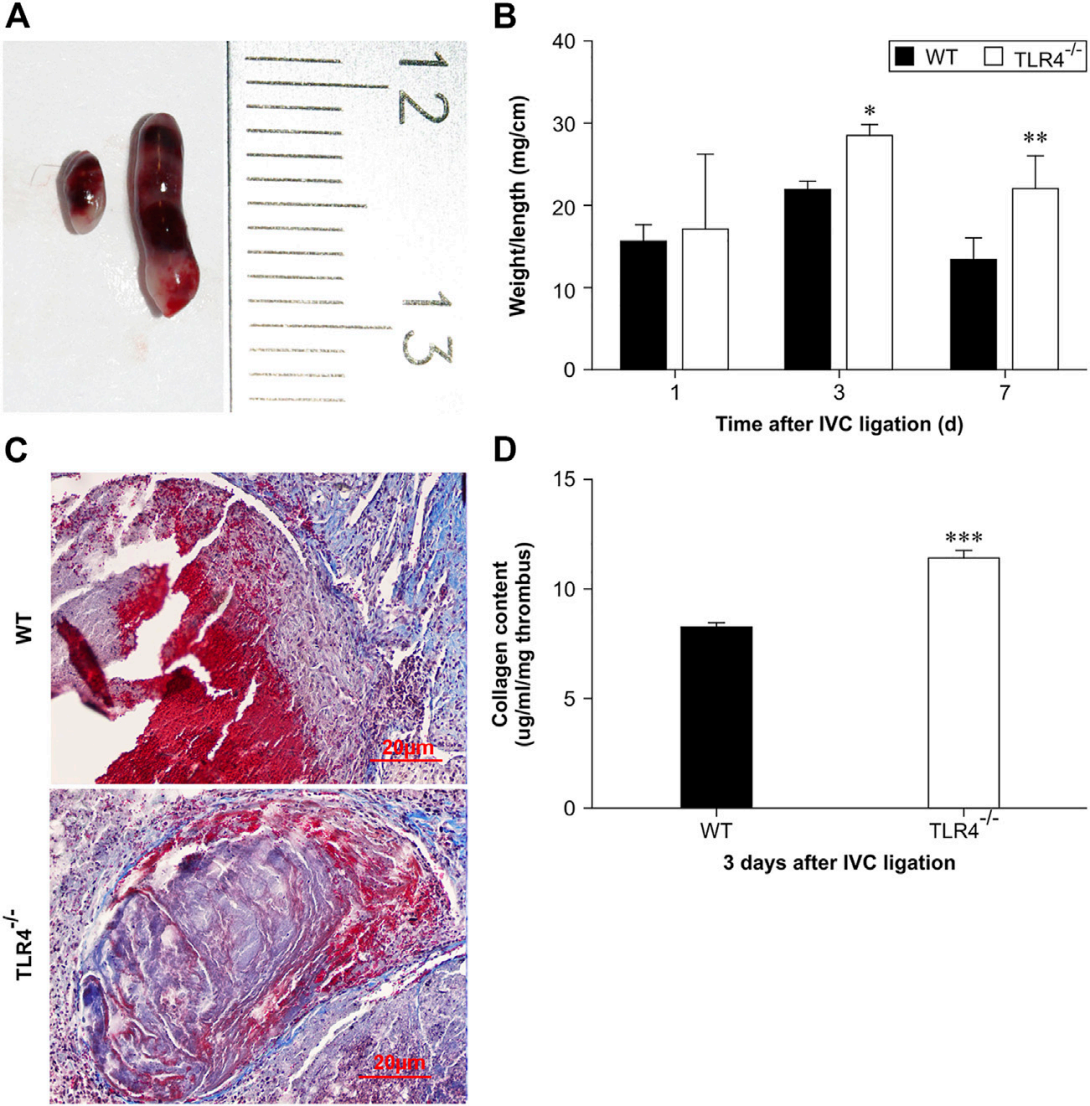
Stasis-induced deep vein thrombus in WT and *Tlr4−/−* mice. **(A)** Macroscopic appearance of intravenous thrombi in WT (left) and Tlr4−/− mice (right) 3 days after the IVC ligation. Representative results from 6 independent animals are shown. **(B)** The thrombus weight-to-length ratios of WT and *Tlr4−/−* mice at the indicated time intervals after IVC ligation. Data are mean ± SD (*n* = 6). *p < 0.05, **p < 0.01, *Tlr4−/− versus* WT. **(C)** Masson staining showed the distribution of collagen in the venous thrombus in WT and *Tlr4−/−* mice at 3 days after IVC ligation (scale bar = 20 µm). **(D)** Intrathrombotic collagen content in WT and *Tlr4−/−* mice at 3 days after IVC ligation. Data represent mean ± SD (*n* = 6). ***p < 0.001, *Tlr4−/− versus* WT.

To investigate the functions of TLR4 in VTE, we constructed a stasis-induced DVT animal model by performing IVC ligation in C57BL/10ScnJ and C57BL/10J mice. 3 days after venous ligation, venous thrombosis was formed in both wild-type and TLR4-deficient mice (Figure 1A). Especially, at 3 and 7 days after ligation, the weight-to-length ratio of venous thrombosis increased in C57BL/10ScnJ mice compared to C57BL/10J mice (*n* = 10–12, *p* < 0.05, *p* < 0.01, Figure 1B). Histopathological analysis of venous thrombi stained with Masson in both WT and Tlr4−/− mice after 3 days of IVC ligation (Figure 1C). As intrathrombotic collagen content can reflect thrombus mass, we separated the thrombi from the vein wall and measured intrathrombotic collagen content using biochemical methods. When compared to WT mice, the Tlr4−/− mice had a higher intrathrombotic collagen concentration (3 days, *n* = 6, *p* < 0.001, Figure 1D). These observations suggested that the loss of TLR4 might impair the resolution of stasis-induced venous thrombosis.

### Tlr4−/− mice had decreased leukocyte recruitment and MCP-1 expression in the thrombus

**FIGURE 2:**
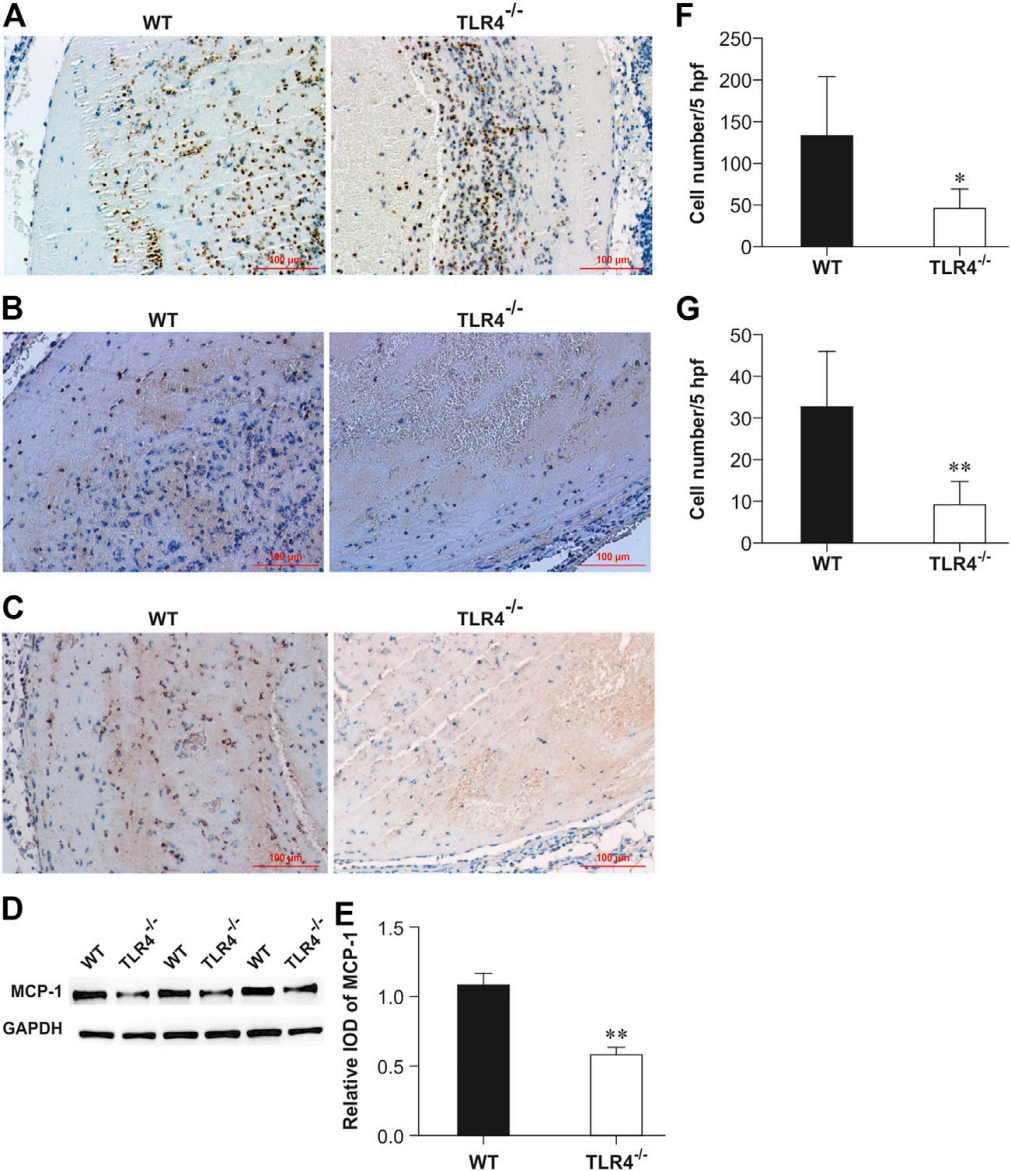
Enumeration of intrathrombotic leukocytes **(A,B)** and expression of MCP-1 protein **(C,D)** in WT and *Tlr4−/−* mice. Immunohistochemical analysis was performed using anti-myeloperoxidase pAbs **(A)**, anti-F4/80 mAbs **(B)**, and anti-MCP-1 mAbs **(C)** in venous thrombi from WT and *Tlr4−/−* mice at 3 days after IVC ligation (scale bar = 100 µm). **(D)** Western blotting analysis of MCP-1. Representative results from 3 independent experiments are shown. **(E)** The ratios of MCP-1 to GAPDH were densitometrically calculated. All values represent the mean ± SD (*n* = 3). **p < 0.01, *Tlr4−/− versus* WT. Representative results from 3 independent experiments are shown. The numbers of neutrophils **(F)** and macrophages **(G)** were determined as described in the methods in 5 high-power fields (hpf). All values represent the mean ± SD (*n* = 6). **p < 0.01, *Tlr4−/− versus* WT.

To investigate TLR4’s function in venous thrombosis resolution, thrombus from WT and Tlr4−/− mice were harvested respectively for immunohistochemical staining of anti-myeloperoxidase and anti-F4/80 at 3 days after IVC ligation. Relative to WT mice, Tlr4−/− animals showed noticeably fewer myeloperoxidase-positive and F4/80-positive cells (*n* = 5-6, *p* < 0.01, Figures 2A, F; *n* = 5-6, *p* < 0.01; Figures 2B, G). We also evaluated MCP-1 expression in the venous thrombus by immunohistochemistry and western blot. Tlr4−/− animals had considerably fewer MCP-1 positive cells and less MCP-1 expression than WT mice (*n* = 5-6, *p* < 0.01, Figures 2C–E). These results suggest that TLR4-mediated MCP-1 expression might be related to leukocyte infiltration into the venous thrombosis.

### Lack of TLR4 reduced MMP-9 activities and expression

**FIGURE 3:**
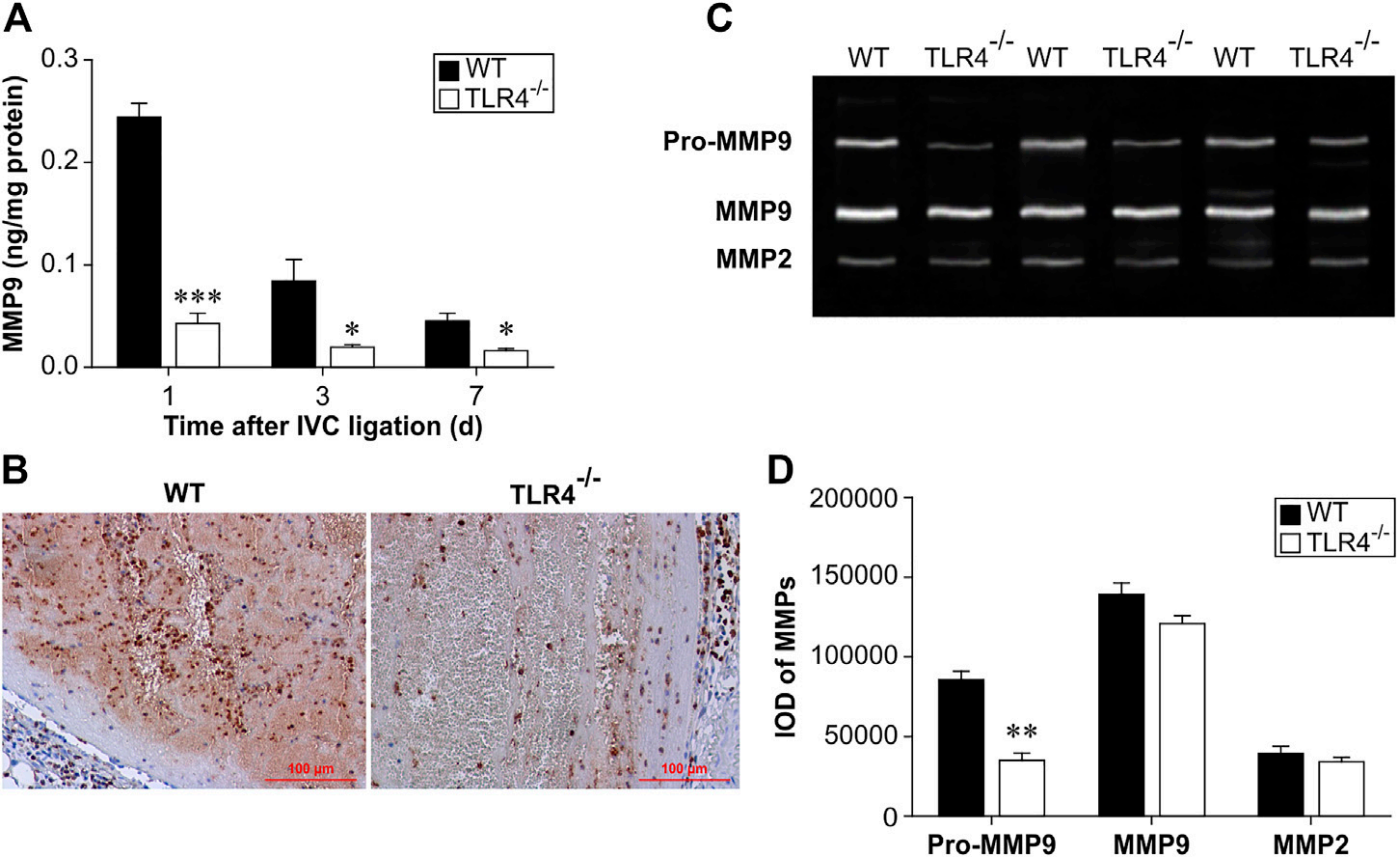
Protein expression and activity of MMP-9 in WT and *Tlr4−/−* mice. **(A)** MMP-9 protein expression in the thrombus measured by ELISA at 3 time points (1, 3, and 7 days after IVC ligation). All values represent the mean ± SD (*n* = 6). *p < 0.05, ***p < 0.001, *Tlr4−/− versus* WT. **(B)** Immunohistochemical analysis of MMP-9 in the thrombus at 3 days after IVC ligation (scale bar = 100 µm). **(C)** Intrathrombotic MMP-2 and MMP-9 activities were measured by gelatine gel zymography as described in the materials and methods. Representative gel images from 3 independent experiments are shown. **(D)** Semi-quantitative analysis of the bands shown in **(C)**. All values represent the mean ± SD (*n* = 3). **p < 0.01, *Tlr4−/− versus* WT.

MMP-9 levels in thrombus homogenates were considerably lower in Tlr4−/− mice than in WT mice at 1, 3, and 7 days following IVC ligation, according to an ELISA analysis (*n* = 6–8, *p* < 0.001, *p* = 0.016, and *p* = 0.017, respectively; Figure 3A). Three days after IVC ligation, Tlr4−/− animals had less MMP-9 positive cells in the thrombus than WT mice, according to immunohistochemical labeling (Figure 3B). Intrathrombotic collagenolysis is an important process for venous thrombosis resolution (Wakefield et al., 2008). MMP-2 and MMP-9, which have molecular weights of 72 kDa and 92 kDa, respectively, are both type IV collagenases. Once activated, they will degrade the collagen types during thrombus resolution. The MMP zymography test was used to assess both MMP-2 and MMP-9 activity in thrombus homogenates. Pro-MMP9 activity was considerably reduced in Tlr4−/− animals compared to WT mice. MMP-2 and MMP-9 activity did not differ between groups in a meaningful way (*n* = 4-5, *p* < 0.01, Figures 3C, D). These findings imply that MMP-9 might be a key player in the thrombus resolution process.

### TLR4 deficiency had no obvious effect on uPA expression

**FIGURE 4:**
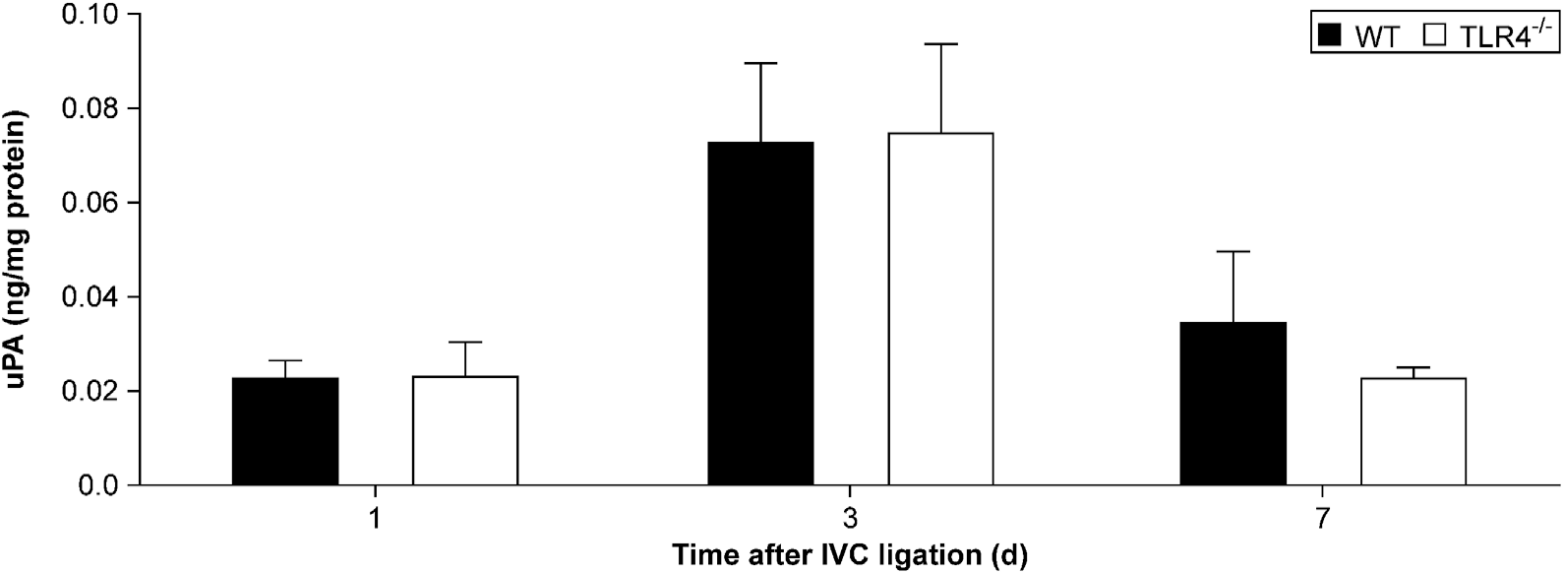
Urokinase plasminogen activator (uPA) protein expression in the thrombus measured by ELISA. There was no significant difference in uPA protein expression between *Tlr4−/−* mice and WT controls at all time points (1, 3, and 7 days) after IVC ligation.

uPA participates in fibrinolysis by converting plasminogen to plasmin and then degrading fibrin. We evaluated the uPA concentration in thrombus homogenates by ELISA in the TLR4-deficient and WT mice at 1, 3, and 7 days after IVC ligation. Between the experimental and control groups at the aforementioned time periods, there was no statistically significant change in uPA expression (*n* = 6–8, *p* > 0.05, Figure 4).

### TLR4 deficiency decreased VEGF expression in later stage of DVT resolution

**FIGURE 5:**
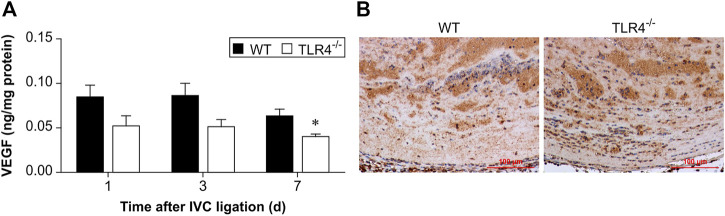
VEGF protein expression in the thrombus. **(A)** VEGF protein expression in the thrombus was measured by ELISA at 3 time points (1, 3, and 7 days after IVC ligation) in WT and *Tlr4−/−* mice. All values represent the mean ± SD (*n* = 6). *p < 0.05, *Tlr4−/− versus* WT. **(B)** Immunohistochemical analysis of VEGF in the thrombus at 3 days after IVC ligation (scale bar = 100 µm).

By controlling angiogenesis, VEGF is crucial in thrombus recanalization. So, at 1, 3, and 7 days following IVC ligation, VEGF concentration in thrombus homogenates was assessed by ELISA. As shown in Figure 5, VEGF expression between the WT and TLR4-deficient mice did not differ significantly at 1 and 3 days after IVC ligation, but the difference was significant at 7 days after IVC ligation (*n* = 5-6, *p* = 0.179, *p* = 0.096, *p* = 0.015, Figure 5A). At 3 days following IVC ligation, immunohistochemical staining confirmed that there was no significant difference statistically in VEGF expression comparing WT and Tlr4−/− animals (Figure 5B).

### TLR4 deficiency decreased IFNβ and MCP-5 expression in the late stage of DVT resolution

**FIGURE 6:**
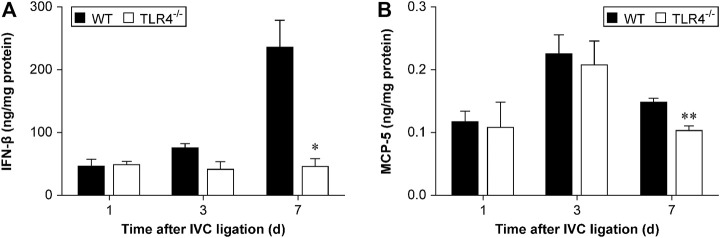
IFNβ and MCP-5 protein expression in the thrombus measured by ELISA. **(A)** IFNβ and **(B)** MCP-5 protein expression in the thrombus of WT and *Tlr4−/−* mice measured by ELISA at 3 time points (1, 3, and 7 days) after IVC ligation. All values represent the mean ± SD (*n* = 6). *p < 0.05, *Tlr4−/− versus* WT.

TLR 4 induces expression of cytokines, chemokines, and other transcription factors through the stimulation of NF-κB and interferon regulatory factor 3 (IRF3) (Palsson-McDermott and O'Neill, 2004). IRF3 activation can stimulate IFNβ expression and induce the phosphorylation of STAT1α/ββ subsequently (Toshchakov et al., 2002). To investigate the function of the TLR4-IRF3-IFNβ-STAT1α/β axis on venous thrombosis resolution, we evaluated IFNβ and MCP-5 expression by ELISA in Tlr4−/− and WT mice at 1, 3, and 7 subsequent days after IVC ligation. IFNβ expression did not differ significantly between Tlr4−/− mice and WT mice at 1 and 3 days following IVC ligation, but it varied significantly at day 7 (*n* = 5-6, *p* = 0.715 and *p* = 0.068, *p* = 0.010; Figure 6A). Although IFNβ expression was gradually increased in WT mice, no significant changes over time were obtained in Tlr4−/− mice. Similarly, the trend of MCP-5 expression and IFNβ were consistent in the two groups (*n* = 5-6, *p* = 0.624 and *p* = 0.855, *p* = 0.007; Figure 6B).

### TLR4 deficiency decreased MMP-9 and MCP-1 expression in macrophages

**FIGURE 7:**
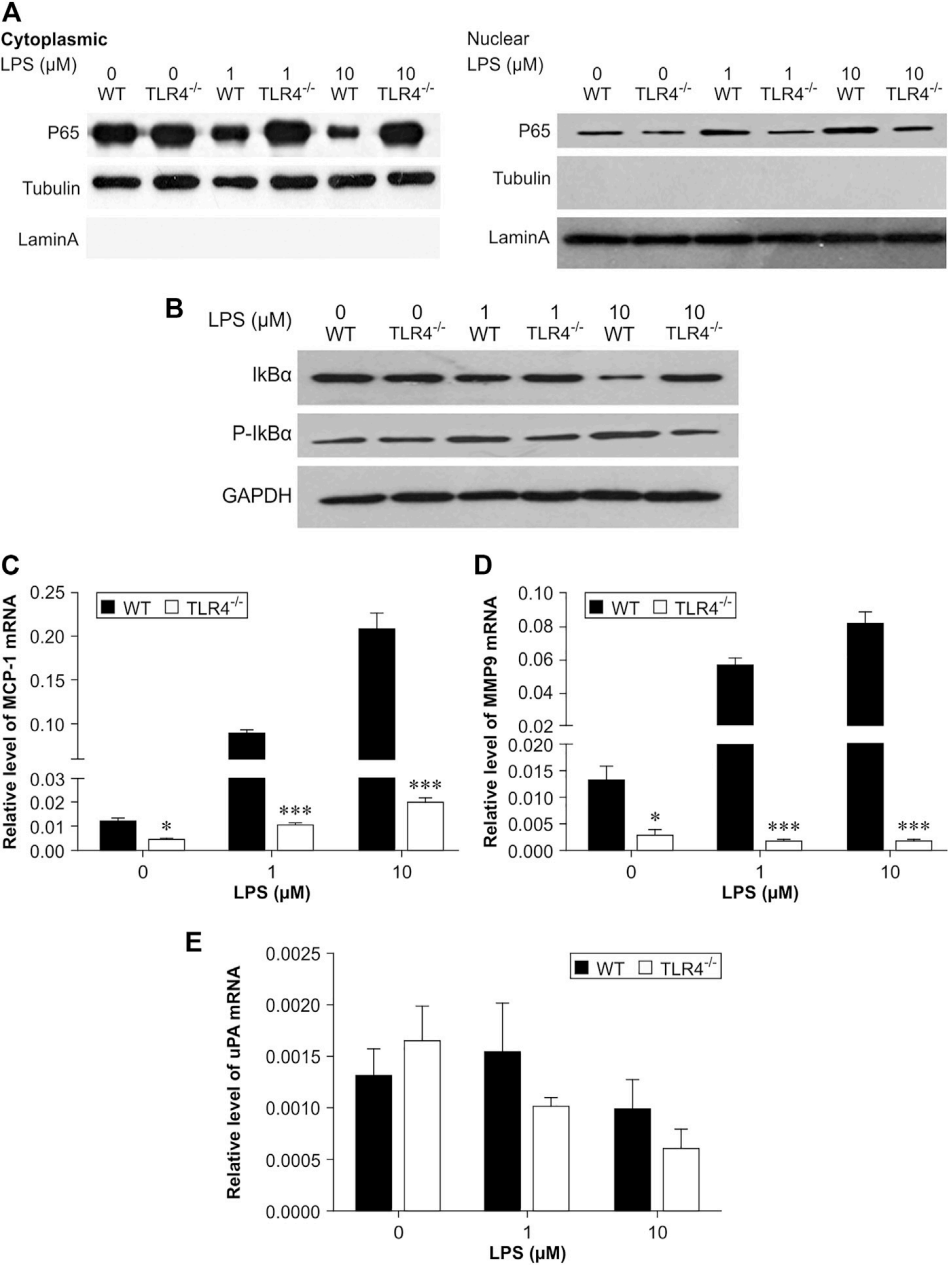
Critical role of NF-κB in lipopolysaccharide (LPS)-induced transcription of *MCP-1* and *MMP-9* in peritoneal macrophages. **(A,B)** Representative results of western blot analysis for p65 **(A)** protein levels and phosphorylation of IκBα **(B)**. Quantitative results of p65, IκBα, and phosphorylation of IκBα protein levels, which were normalized to the internal control tubulin, lamin A, or GAPDH. **(C–E)** *MCP-1* **(C)**, *MMP-9* **(D)**, and *uPA* **(E)** mRNA expression measured by real-time PCR. The results were normalized to the number of β-actin transcript copies. Data represent mean ± SD (*n* = 3), *p < 0.05, **p < 0.01, ***p < 0.001, *Tlr4−/− versus* WT.

In experimental animal models, the accumulation of macrophages within a thrombus is very important in thrombus resolution. Our *in vivo* observations suggested that TLR4 deficiency decreased MMP-9 and MCP-1 expression in the venous thrombus (Figures 2, 3). Meanwhile, the number of macrophages infiltrating the thrombus in Tlr4−/− mice was significantly reduced. To determine the TLR4 effect on macrophage-induced transcription of MCP-1, MMP-9 and uPA, we collected peritoneal macrophages from both WT and Tlr4−/− mice. Phosphorylation and degradation induced by macrophages treated with LPS were dose-dependent. It prompted p65 nuclear translocation and phosphorylation of IκBα to accelerate the transcription of MMP-9 and MCP-1 in WT macrophages, but didn't occur in TLR4-deficient macrophages (*n* = 4-5, *p < 0.05, **p < 0.01, ***p < 0.001, Figure 7). These findings showed that TLR4 activation can induce both MMP-9 and MCP-1 transcription via NF-κB, but TLR4 deficiency impairs this process.

## Discussion

TLR4 is a crucial factor linking the processes of inflammation and innate immunity. TLR4 regulates immune response not only against microbial components in infectious disease but also against endogenous ligands in the sterile inflammation process. TLR4 has been shown to be involved in sterile inflammation reactions such as ischaemia-reperfusion injury in myocardial infarction, hepatic, renal, and intestinal ischaemia. A recent experimental study suggested that venous thrombosis resolution is a sterile inflammation process (Ripplinger et al., 2012). Thus, these data motivated us to consider whether TLR4 might play an important role in thrombosis resolution. In this project, we found that TLR4 is included in thrombus resolution; TLR4 deficiency impaired DVT resolution through downregulating the expression of MCP-1 and MMP-9, which was possibly caused by depriving the TLR4/NF-κB pathway.

Neutrophils and macrophages are successively injected into the thrombus as part of the DVT resolution process. In a DVT rat model, neutropenia impaired the lysis of venous thrombosis (Varma et al., 2003). However, neutrophil depletion in mice does not impair DVT resolution (Henke et al., 2004). Intrathrombus macrophage accumulation is the hallmark of the later stage of DVT resolution (Wakefield et al., 2008; Saha et al., 2011). An increased number of macrophages in the thrombus enhances DVT resolution (Ali et al., 2006). Normal DVT resolution, primarily relying on macrophages, involves CXCR2-mediated neovascularization, collagen conversion, and fibrinolysis using a CXCR2^−/−^ mouse DVT model (Henke et al., 2004). CCR2 receptor activation is important for monocyte recruitment into the venous thrombus. Targeted CCR2 deletion impairs DVT resolution by affecting monocyte infiltration, neovascularization, and MMP-2 and MMP-9 activity (Ali et al., 2006). CCR2 is a cell surface receptor that binds MCP-1. MCP-1 treatment increases the mechanization and dissolution of the thrombus (Ali et al., 2006). In our study, the numbers of infiltrated neutrophils and macrophages decreased and the expression of MCP-1 was lower in TLR4-deficient mice than WT mice. This suggests that TLR4 deficiency affects MCP-1 expression, thereby reducing the infiltration of both neutrophils and macrophages.

DVT resolution is similar to wound healing processes which involve collagen and MMP. MMP production and activity can be regulated at various levels, including gene transcription, endogenous repression, and pro-MMP activation (Hobeika et al., 2007). MMPs are an inactive precursor generated in cells, secreted extracellularly to be activated. Enhanced MMP-9 gene expression and activity accelerated DVT resolution in later phase in IFN-γ^−/−^ mice (Nosaka et al., 2011). A study performed in neutropenic rats suggested that a decrease in intrathrombus MMP-9 gelatinase activity is related to impairment of DVT resolution in early phase (Henke et al., 2006). Here, we showed that TLR4-deficient mice had significantly lower levels of MMP-9 expression than TLR4-intact mice at 1, 3, and 7 days after IVC ligation. Lower gelatinase activity of pro-MMP-9 in TLR4-deficient mice than mice with TLR4-intact. As a result, deletion of TLR4 seems to make it more difficult to remove thrombi through decreasing MMP-9 production and activity.

The consequences of thrombus clearance are vascular recanalization and remodeling of the venous wall (Varma et al., 2004). In our study, there are statistically significant differences in DVT resolution between Tlr4−/− mice and WT mice at 3 and 7 days after IVC ligation. The DVT resolution in Tlr4−/− mice was reduced compared to those from WT mice. ELISA of VEGF indicated significant differences in VEGF expression in thrombus homogenates at 7 days after IVC ligation. This result suggests that VEGF may be involved in late DVT resolution.

Plasmin is a major fibrinolytic protease that participates in DVT resolution. Circulating tissue PA (tPA) and uPA converts plasminogen to plasmin. Both tPA and uPA are secreted in a single-chain form by endothelial cells or monocytes/macrophages, and activated by binding into duplexes. Mouse models of uPA^−/−^ and tPA^−/−^ IVC thrombosis showed that venous thrombus resolution was associated with uPA, but not affected by tPA deficiency (Singh et al., 2003). Here, we evaluated uPA expression in the thrombus homogenate by ELISA at 1, 3, and 7 days after IVC ligation. The expression of uPA was not significantly change between TLR4-deficient and TLR4-intact mice, suggesting that uPA is dispensable in TLR4-related venous thrombolysis.

Similar to the results of VEGF, the expression of IFNβ and MCP-5 in the thrombus homogenates of TLR4-deficient mice and WT mice was significantly different only at 7 days after IVC ligation. These data suggested that the TLR4/TRIF pathway is not necessary for TLR4-dependent DVT resolution. We next investigated the relation of TLR4 deletion on NF-κB activation *in vitro*. LPS treatment appears to significantly increase MMP-9 and MCP-1 transcription in peritoneal macrophages through TLR4/NF-κB.

## Conclusion

This study indicates that DVT resolution was significantly different between Tlr4−/− and wild type mice. Compared with wild mice, Tlr4−/− mice had increased weight-length ratio and collagen content in venous thrombus. Tlr4−/− mice had decreased in MCP-1 expression and leukocyte recruitment and reduced expression and activity of MMP-9 in venous thrombus. After 7 days of IVC ligation, VEGF, IFNβ, and MCP-5 protein expression were decreased in venous thrombus from Tlr4−/− mice. *In vitro* experiments showed that activation of TLR4 receptors in peritoneal macrophages from wild-type mice was able to promote p65 nuclear transfer and phosphorylation of IκBα and accelerate the transcription of MMP-9 and MCP-1 compared with peritoneal macrophages from Tlr4−/− mice. In conclusion, TLR4 is involved in venous thrombosis resolution through NF-κB pathway. Loss of TLR4 in mice impairs the process.

## Limitations

DVT resolution is a sterile inflammatory process. This study investigated the effect of TLR4 on DVT resolution. *In vitro* experiments, TLR4 affected the transcription of downstream MCP-1 and MMP-9 through activation of the NF-κB pathway. We also need to observe the transcriptional effects of NF-κB pathway inhibitors on MCP-1 and MMP-9.

This study found that TLR4 deficiency affected numerous effector molecules such as MCP-1 and MMP-9 as well as VEGF, IFNβ, and MCP-5, 7 days after venous ligation. Animal experiments are needed to further verify which molecules play key roles in DVT resolution. TLR4 is activated in response to endogenous ligands. The authors will continue to explore the effects of endogenous ligands on DVT resolution via TLR4.

## Data Availability

The original contributions presented in the study are included in the article/supplementary material, further inquiries can be directed to the corresponding author.
